# Riboflavin, vitamin B2, attenuates NLRP3, NLRC4, AIM2, and non-canonical inflammasomes by the inhibition of caspase-1 activity

**DOI:** 10.1038/s41598-020-76251-7

**Published:** 2020-11-05

**Authors:** Huijeong Ahn, Geun-Shik Lee

**Affiliations:** Laboratory of Inflammatory Diseases, Department of Physiology, College of Veterinary Medicine and Institute of Veterinary Science, Kangwon National University, Chuncheon, Gangwon 24341 Republic of Korea

**Keywords:** Inflammation, Inflammasome, Interleukins

## Abstract

Riboflavin is commonly taken as a nutritional supplement, and it converts to coenzymes during the process of energy production from carbohydrates, fats, and proteins. Although riboflavin is considered to be an anti-inflammatory vitamin because of its antioxidant properties, the effects of riboflavin on inflammasome have been not reported. Inflammasome, a cytosolic surveillance protein complex, leads to the activation of caspase-1, cytokine maturation, and pyroptosis. In the present study, riboflavin attenuated the indicators of NLRP3 inflammasome activation in macrophages, such as the maturation and secretion of interleukin (IL)-1β, IL-18, and caspase-1; the formation of Asc pyroptosome; and the cleavage of gasdermin D. In addition, the oral and peritoneal administration of riboflavin inhibited the peritoneal production of IL-1β and IL-18 in a mouse model. Mechanistically, riboflavin prevented mitochondrial perturbations, such as mitochondrial ROS production and mitochondrial DNA release, which trigger the NLRP3 inflammasome assembly. Riboflavin was further confirmed to disrupt the activity of caspase-1, and it also inhibited the AIM2, NLRC4, and non-canonical inflammasomes. Therefore, riboflavin has both an antioxidant effect and an anti-inflammasome property that regulates the inflammatory response.

## Introduction

Vitamin B, a class of water-soluble vitamins, acts as a cofactor or coenzyme in cellular metabolism. Although a class of B vitamins shares a common name, such as B1, B2, B3, B5, and B6, their chemical structures are different. Vitamin B2 (riboflavin) is a precursor of flavin adenine dinucleotide (FAD) and flavin mononucleotide (FMN), and is involved in energy release in the electron transport chain, the tricarboxylic acid cycle, and the catabolism of fatty acids^1^. Riboflavin should be supplied by food or the biosynthesis of intestinal microflora because it cannot be synthesized by animals, including humans^2^. A riboflavin deficiency causes inflammation in the mouth or around the mouth, such as stomatitis, cheilosis, glossitis, and dermatitis, which might increase the risk of some tumors^3^. For therapeutic purposes, riboflavin is supplied to prevent migraines and offer protection from neurological disorders, such as Parkinson’s diseases and multiple sclerosis^3^.

Riboflavin has been reported to maintain the physiological homeostasis, including the immune system^2^. The phagocytosis, proliferation, and apoptotic cell death of innate immune cells were differently regulated by the supplementation or deficiency of riboflavin^2^. In addition, riboflavin changes the production of inflammatory mediators, such as tumor necrosis factor (TNF) α, high-mobility group protein 1 (HMG-1), nitrogen oxide (NO), heat-shock protein (Hsp) 72, interleukin (IL)-1β, monocyte chemoattractant protein 1 (MCP1), interferon (INF) γ, and IL-10^2,4,5^. In animal models, the injection of riboflavin ameliorates the death resulting from sepsis and bacterial infection^5,6^. Inflammasome is also tightly involved in cytokine maturation and sepsis^7^. Inflammasome, an intracellular multiprotein complex, is assembled by encountering the endogenous and pathogenic danger signals. As a result of the inflammasome assembly, the activated caspase-1 (Casp1) leads to cytokine maturation and inflammatory cell death, pyroptosis^8^. Inflammasomes are classified by sensor proteins, such as nucleotide-binding oligomerization domain-like receptor (NLR) pyrin domain-containing protein 3 (NLRP3), NLR caspase activation, and recruitment domain-containing protein 4 (NLRC4), absent in melanoma 2 (AIM2) and caspase-11 in rodents (caspase-4/5 in humans, also called as non-canonical inflammasome)^8^. NLRP3 inflammasome mediates several metabolic, senile, pathogenic, and genetic disorders^9^. NLRC4 and AIM2 inflammasomes are activated mainly by pathogens, and the non-canonical inflammasome is a key factor in septic shock^10,11^.

Although the anti-inflammatory function of riboflavin has been studied progressively, few studies have examined the role of riboflavin on inflammasome. In the current study, the effects of B vitamins on the activation of NLRP3 inflammasome in macrophages were screened. The effects of riboflavin as a candidate anti-NLRP3 molecule were examined by several indicators of the NLRP3 inflammasome activation and an animal model. Furthermore, the inhibitory properties of riboflavin on NLRP3 inflammasome were expanded to the disruption of other inflammasomes (AIM2, NLRC4, and non-canonical inflammasomes) based on the inhibition of riboflavin on the activity of Casp1.

## Results

### Vitamin B regulates the activation of NLRP3 inflammasome

**Figure 1:**
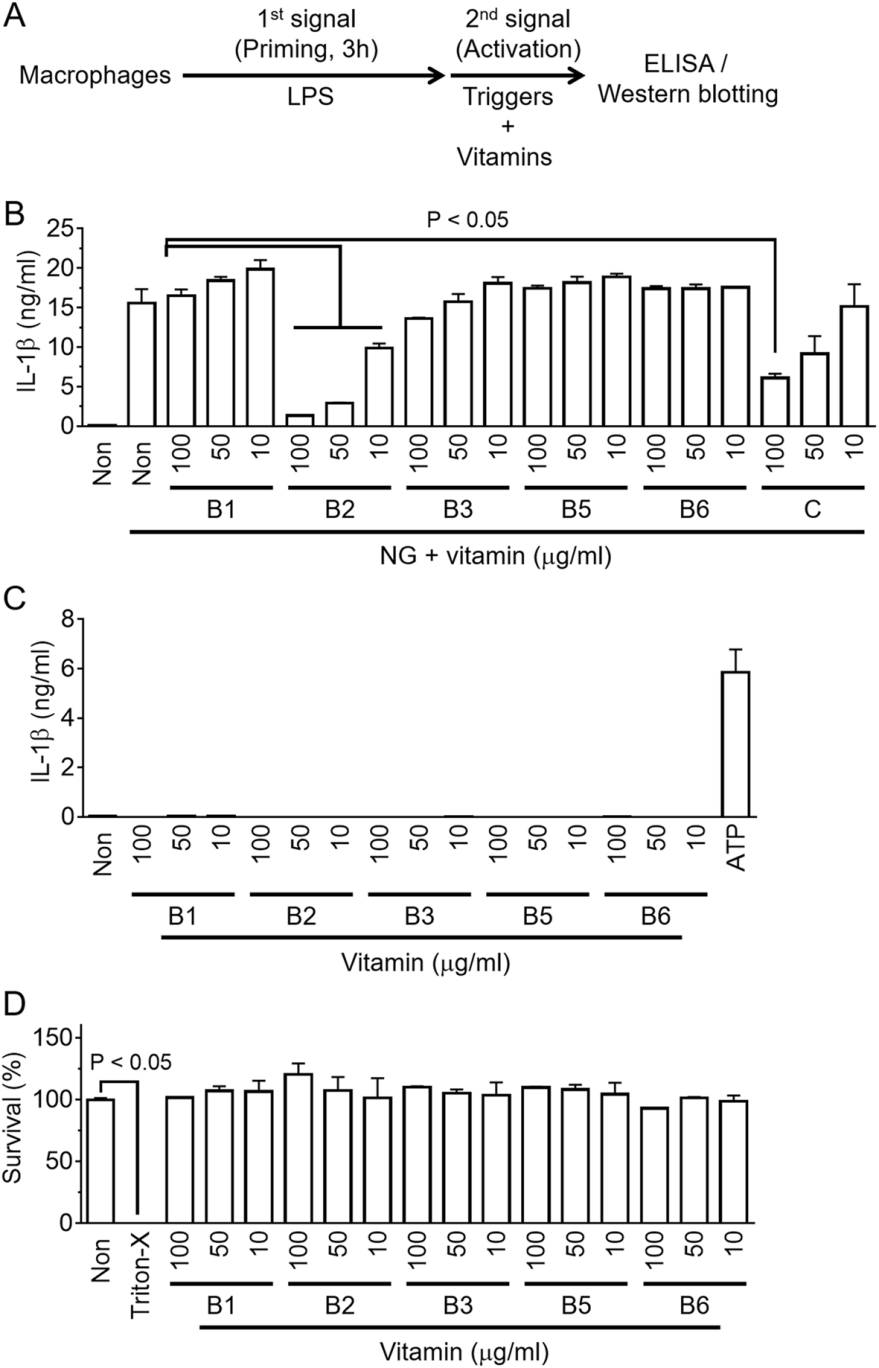
Effects of vitamin B on NLRP3 inflammasome activation. (**A**) Schematic diagram of the experimental process of inflammasome activation. Macrophages, such as BMDMs or PMA-treated THP-1 cells, were primed with LPS for 3 h, and the inflammasome activation was then triggered in the presence of a vitamin. The indicators of the inflammasome activation were analyzed by ELISA or Western blotting. (**B**) LPS-primed BMDMs activated the NLRP3 inflammasome with NG with/without a vitamin as indicated. The secretion of IL-1β was measured by ELISA. (**C**) LPS-primed BMDMs were treated with a vitamin, as indicated without the inflammasome triggers. The ATP treated group was used as a positive control. (**D**) LPS-primed BMDMs were treated a vitamin, as indicated for 24 h, and the cytotoxicity was analyzed. The survival rates of the non-treated cells were set to 100%, and the cells treated with triton X-100 (Triton-X, 0.01%) were regarded as 0% viability. The bar graph presents the mean ± SD with at least two independent experiments.

Murine bone marrow-derived macrophages (BMDMs) were primed with lipopolysaccharide (LPS) and subjected to a nigericin (NG) treatment to trigger the NLRP3 inflammasome assembly in the presence of vitamin B1, B2, B3, B5, B6 (pyridoxine), and C, as shown in Fig. 1A. The secretion of IL-1β, an indicator of inflammasome activation, was increased by the NG treatment, as expected (Fig. 1B). Interestingly, the releases of IL-1β were attenuated by vitamin B2 (riboflavin) in a dose-dependent manner, whereas the other B vitamins had little effect. Similar to a previous study^12^, vitamin C also inhibited the release of IL-1β of NLRP3 inflammasome activation. In addition, this study tested the effects of the B vitamins on inflammasome activation without an inflammasome trigger in LPS-primed BMDMs (Fig. 1C). As a result, one vitamin B alone did not lead to IL-1β secretion resulting from inflammasome activation, whereas ATP, an NLRP3 trigger, stimulated IL-1β secretion, as expected. The current B vitamins did not induce any cytotoxicity in the BMDMs (Fig. 1D). Overall, riboflavin is a vitamin that disrupts NLRP3 inflammasome activation.

### Riboflavin inhibits the NLRP3 inflammasome assembly

**Figure 2:**
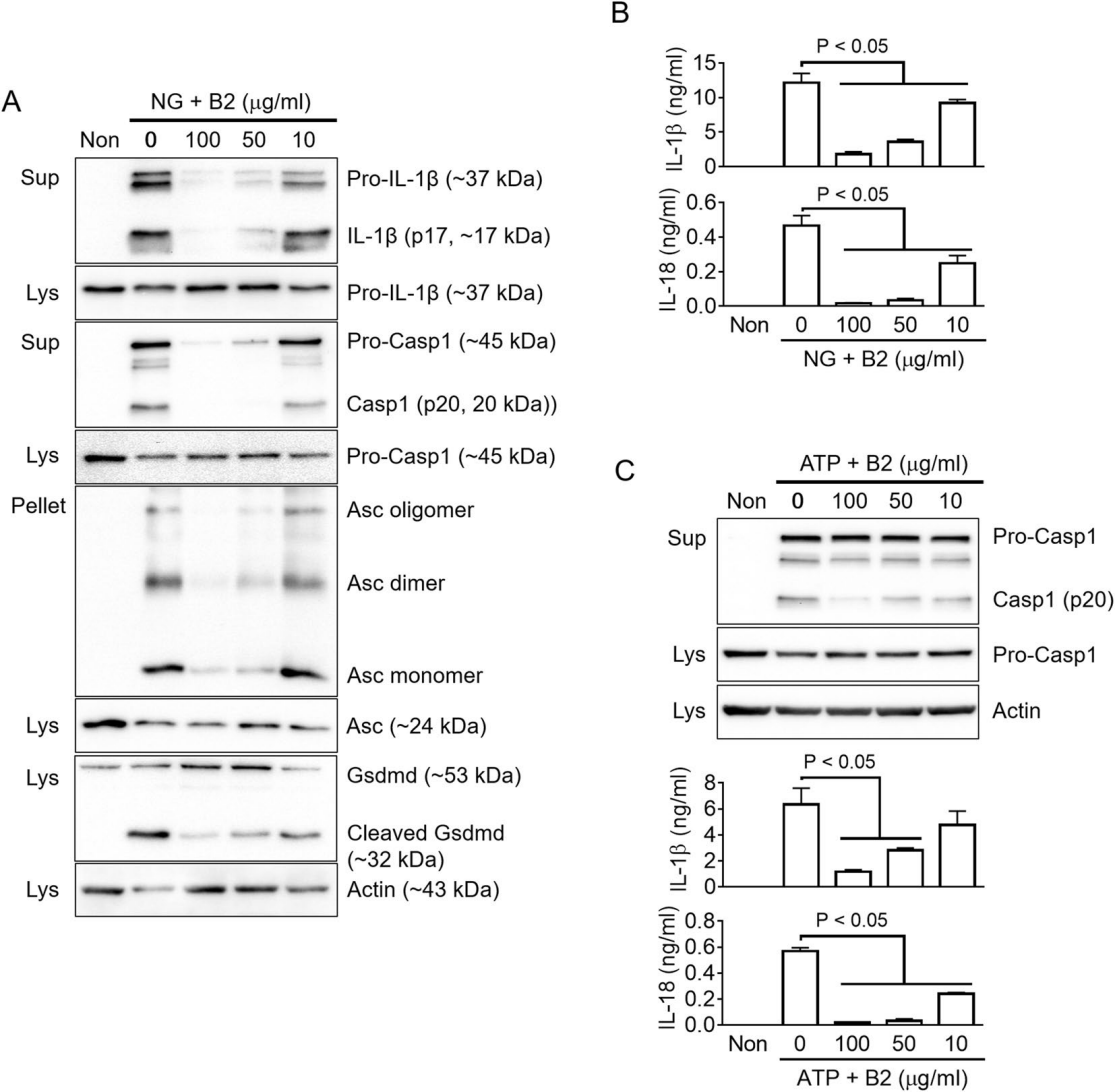
Effects of riboflavin on the activation of NLRP3 inflammasome. (**A**) LPS-primed BMDMs were treated with NG with/without riboflavin (B2). The maturation and secretion of IL-1β and caspase-1 (Casp1), the formation of Asc oligomerization, and the cleavage of Gsdmd were analyzed by immunoblotting as indicated. (**B**) The secretion of IL-1β and IL-18 were measured by ELISA using the supernatant of panel A. (**C**) LPS-primed BMDMs were treated with ATP in the presence of B2. The maturation and secretion of Casp1 were analyzed by immunoblotting, and the IL-1β and IL-18 releases were measured by ELISA. Sup, cellular supernatant; Lys, cellular lysate; Pellet, cross-linked pellet. All immunoblot data shown are representative of at least two independent experiments. The bar graph presents the mean ± SD with at least two independent experiments.

Although the secretion of IL-1β (p17) is the most well-accepted indicator of inflammasome activation, other readouts, such as the secretion of Casp1 (p20/p10) and IL-18, the formation of ASC pyroptosome and the cleavage of gasdermin D (Gsdmd), were also observed to affect the assembly of inflammasomes^9,13^. To confirm the anti-NLRP3 inflammasome property of riboflavin, the effects of riboflavin on the indicators were tested by immunoblotting in NG-treated BMDMs. As shown in Fig. 2A, riboflavin attenuated the secretion of IL-1β (p17) and Casp1 (p20) in a dose-dependent manner. In addition, Asc speck formation and Gsdmd cleavage resulting from NG-mediated NLRP3 inflammasome activation were attenuated significantly by a riboflavin treatment. Similar to the immunoblotting assays, riboflavin decreased the IL-1β and IL-18 secreted from the NG-treated BMDMs, as confirmed in ELISA (Fig. 2B). The other trigger, ATP, was adopted to assemble the NLRP3 inflammasome, and the anti-NLRP3 role of riboflavin in the ATP-treated BMDMs was assessed (Fig. 2C). Riboflavin successfully blocked the secretion of Casp1 (p20), IL-1β, and IL-18. Overall, riboflavin inhibits the activation of NLRP3 inflammasome.

### Riboflavin curbs peritoneal IL-1β and IL-18 production in an animal model

**Figure 3:**
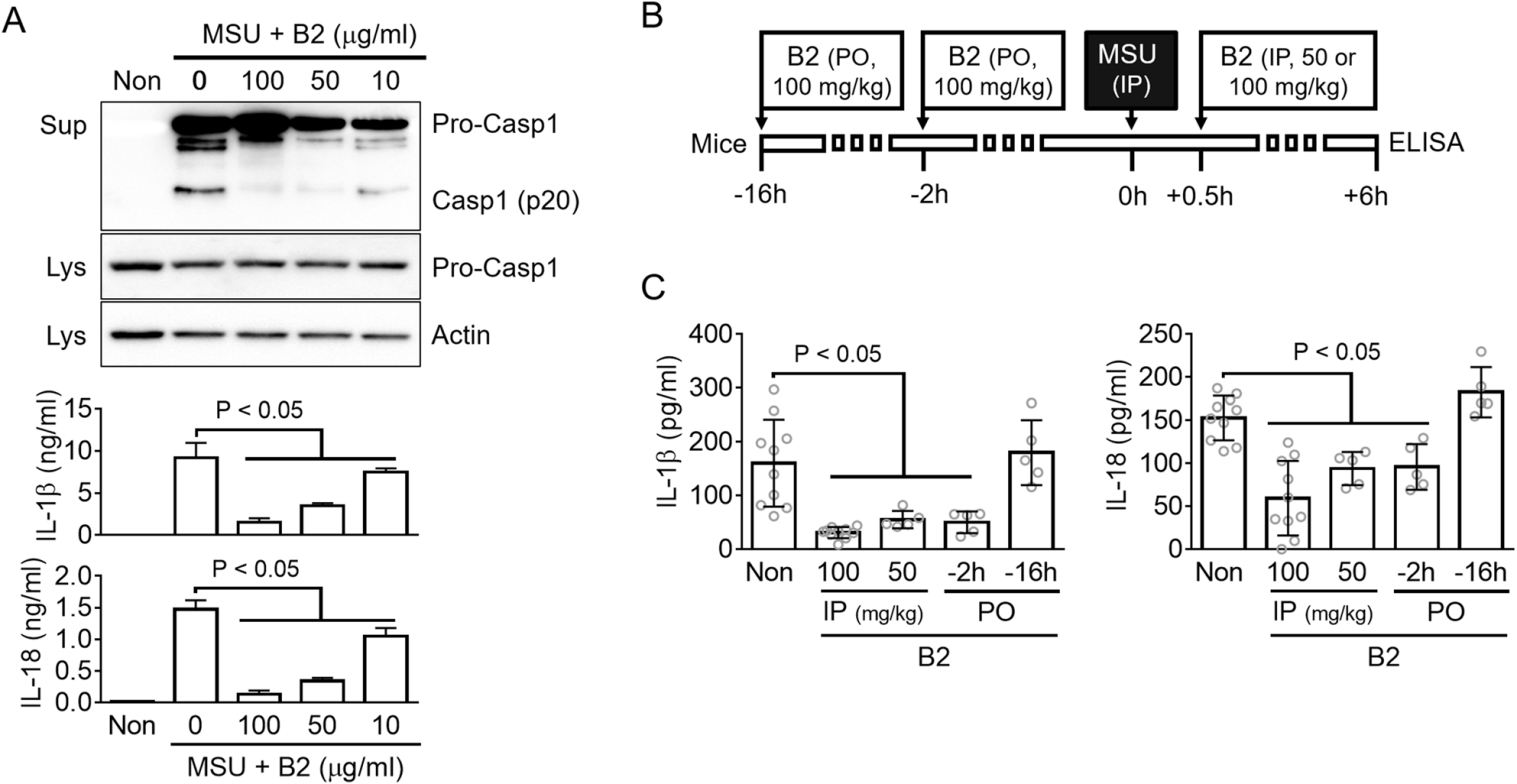
Effects of riboflavin on MSU-induced peritonitis in mice. (**A**) LPS-primed BMDMs were treated with MSU with/without riboflavin (B2). Casp1 maturation was observed by immunoblotting and the release of IL-1β and IL-18 was detected by ELISA. (**B**) The schematic diagram indicates the procedure of an animal experiment. The mice were administered with B2 by oral gavage (per oral, PO), and then injected with MSU. In addition, the MSU-treated mice were injected intraperitoneally (IP) with B2 at the indicated time. (**C**) The IL-1β and IL-18 secretion in the peritoneal lavage was measured by ELISA. Each gray circle indicates the measured value of each mouse and the number of mice. All immunoblot data shown are representative of at least two independent experiments. The bar graph presents the mean ± SD with at least two independent experiments.

A monosodium urate crystal (MSU) is one of the NLRP3 triggers. Moreover, an intraperitoneal injection of MSU in mice provoked the maturation of IL-1β and IL-18 resulting from NLRP3 inflammasome activation^14,15^. First, the effects of riboflavin on the NLRP3 inflammasome in the MSU-treated macrophages were examined (Fig. 3A). Similar to the above result, riboflavin inhibited the secretion of Casp1, IL-1β, and IL-18 resulting from the MSU-mediated NLRP3 inflammasome assembly. Second, this study examined whether riboflavin interrupted the cytokine production in the MSU-induced peritonitis in mice. As shown in Fig. 3B, mice were injected with two doses (50 or 100 mg/kg) of riboflavin at 30 min after the MSU treatment, or fed with 100 mg/kg of riboflavin at 2 h or 16 h by an oral gavage before the MSU injection. Peritoneal lavages were collected at 6 h after MSU administration, and the levels of IL-1β and IL-18 secretion were measured by ELISA. As expected, the MSU injection induced the production of peritoneal IL-1β and IL-18, and the production was attenuated significantly by the intraperitoneal riboflavin injection (Fig. 3C). In addition, the oral administration of riboflavin at 2 h before the MSU injection blocked the secretion of IL-1β and IL-18, whereas the oral gavage of riboflavin at 16 h did not change the levels of cytokine production. Therefore, riboflavin not only inhibits the secretion of IL-1β and IL-18 in MSU-treated macrophages but also MSU-injected mice.

### Riboflavin prevents the perturbation of mitochondria and disrupts the activation of Casp1

**Figure 4:**
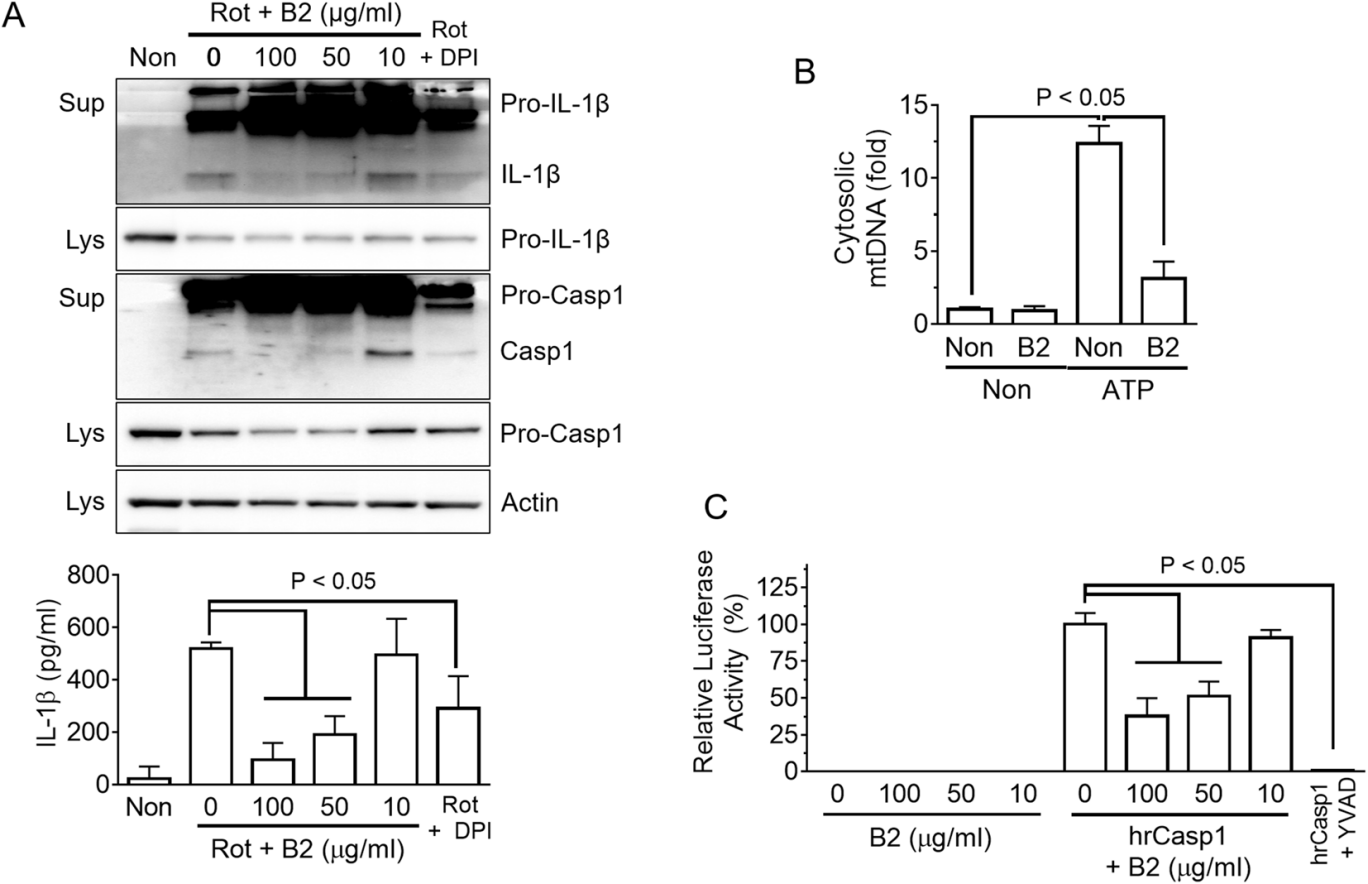
Mechanistic study of the anti-inflammasome property of riboflavin. (**A**) LPS-primed BMDMs were treated with rotenone (Rot) with/without riboflavin (B2). The maturation and secretion of IL-1β and Casp1 were analyzed by immunoblotting and the secretion of IL-1β was analyzed by ELISA. Diphenyleneiodonium chloride (DPI) was used as a ROS scavenger. (**B**) LPS-primed BMDMs were treated with ATP in the presence of B2. The cytosolic release of mitochondrial DNA (mtDNA, cytochrome c oxygenase 1/18S rDNA) was measured by quantitative real-time PCR. (**C**) The activity of human recombinant caspase-1 (hrCasp1) in the presence of B2 was measured using a commercial kit, which is a modified luciferase assay. Briefly, hrCasp1 catalyzes Z-WEHD-aminoluciferin to aminoluciferin, a substrate of luciferase, resulting in the generation of light, which represents the activity of Casp1. Ac-YVAD-CHO (YVAD, the kit supplied) was utilized as a caspase-1 inhibitor. All immunoblot data shown are representative of at least two independent experiments. The bar graph presents the mean ± SD with at least two independent experiments.

NLRP3 triggers perturb the stability of mitochondria, resulting in the production of mitochondrial reactive oxygen species (mtROS) and the release of mitochondrial DNA (mtDNA) into the cytosol, which oxidized further in the cytosol^9,16,17^. Both mtROS and mtDNA resulting from the perturbation of mitochondria are involved tightly in the assembly of NLRP3 inflammasome^9^. In addition, riboflavin acts as an antioxidant to protect against oxidative stress^18^. Therefore, it was hypothesized that riboflavin inhibited NLRP3 inflammasome activation by preventing the perturbation of mitochondria^9,18^. LPS-primed BMDMs were treated with rotenone, which in the presence of riboflavin, disrupted mitochondrial respiration and produced mtROS^17^. Similar to a previous study^16,17^, rotenone elicited the secretion of IL-1β and Casp1 from NLRP3 inflammasome activation, which was blocked by the riboflavin treatment (Fig. 4A). In addition, the effects of riboflavin on the releases of mtDNA in response to NLRP3 triggers were examined (Fig. 4B, Supplementary Fig. S1A). As a result, ATP was provoked to increase the cytosolic mtDNA, which was attenuated by riboflavin. Thus, riboflavin might protect the cells from the mitochondrial damage induced by NLRP3 triggers culminating in the inhibition of NLRP3 inflammasome activation. Next, we treated the effect to riboflavin on the priming step of NLRP3 inflammasome activation when the component and substrate of inflammasome were up-regulated by toll-like receptor (TLR) ligands, such as LPS. BMDMs were treated with riboflavin with/without LPS (Supplementary Fig. S1B). As expected, the level of the NLRP3 and pro-IL-1β proteins were increased by LPS priming, but riboflavin did not change the protein expression. In addition, the transcriptional levels of cytokines, such as *IL-1α, IL-1β, IL-6, IL-10*, and *TNFα* were measured (Supplementary Fig. S1C). As a result, no cytokine was expressed differently by the riboflavin treatment. In addition, the effects of riboflavin on the secretion of pro-inflammatory cytokines, such as TNFα and IL-6, were examined. BMDMs were treated with LPS for 3 h or 6 h, and the cytokines in the media and cellular lysates were measured (Supplementary Fig. S2A). Riboflavin blocked the secretion of TNFα and IL-6 significantly, but did not change the intracellular levels of TNFα, IL-6, and IL-1β. This suggests that riboflavin might change the process of cytokine secretion or cellular permeability. Finally, this study examined the effects of riboflavin on the activity of Casp1 using recombinant human caspase-1 (rhCasp1). As shown in Fig. 4C, riboflavin attenuated the activity of rhCasp1 significantly, as well as the inhibitor of Casp1, Ac-YVAD-CHO. The activity of recombinant mouse caspase-1 was also inhibited by riboflavin (Supplementary Fig. S2B). Overall, riboflavin blocked the activation of NLRP3 inflammasome by inhibiting mtROS production, mtDNA release, and Casp1 activity.

### Riboflavin inhibits the activation of AIM2, NLRC4, and non-canonical inflammasomes

**Figure 5:**
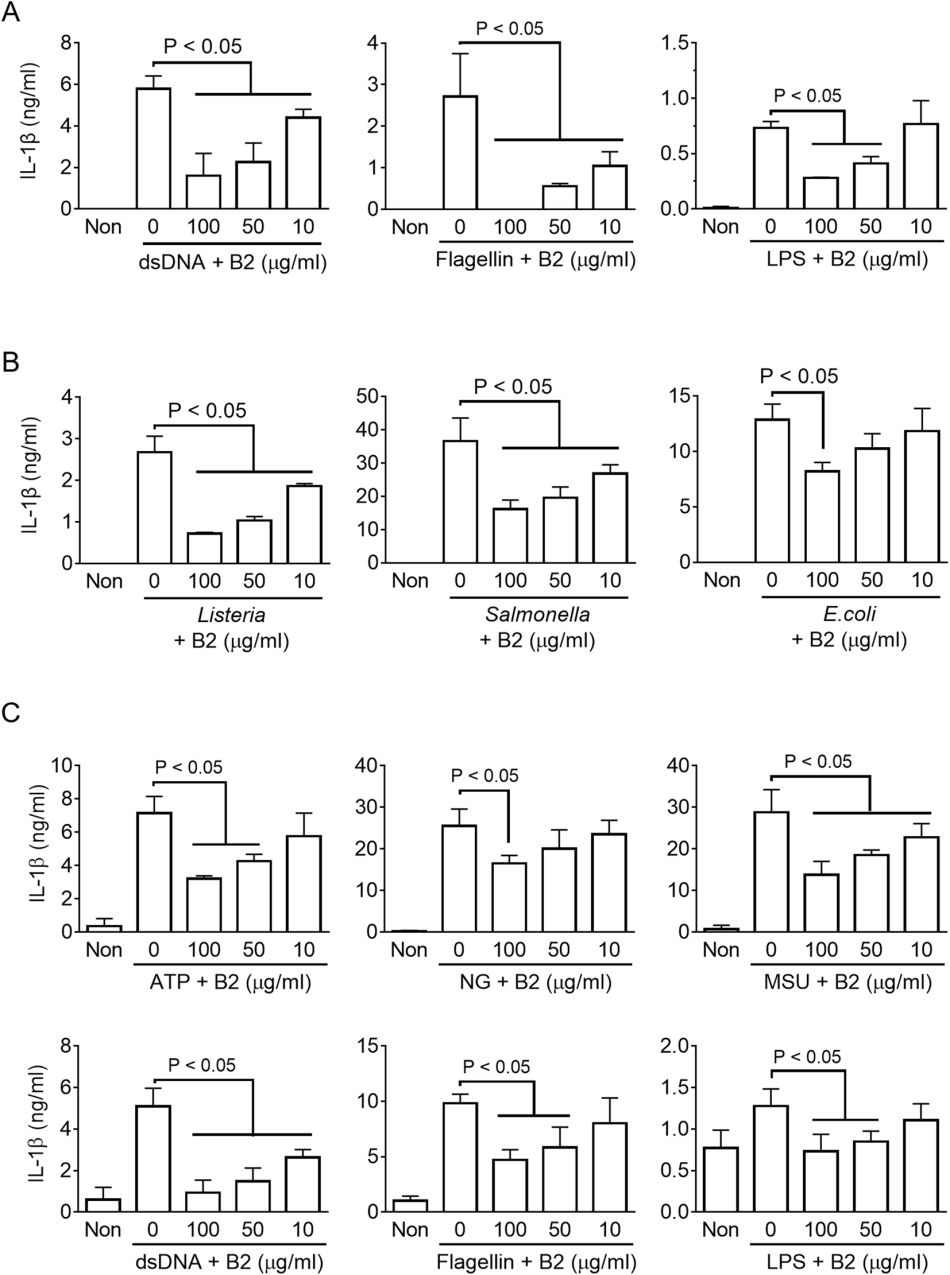
Effects of riboflavin on the activation of the other inflammasomes. (**A**) LPS-primed BMDMs were introduced with dsDNA, flagellin, and LPS into the cytosol to activate AIM2, NLRC4, and non-canonical inflammasomes in the presence of riboflavin (B2). The secretion of IL-1β was measured by ELISA. (**B**) LPS-primed BMDMs were infected with *Listeria*, *Salmonella*, or *E. coli* with/without B2. The release of IL-1β was analyzed by ELISA. (**C**) PMA-treated THP-1 cells were primed with LPS and treated with inflammasome triggers or B2, as indicated. The IL-1β secretions were detected by ELISA. The bar graph presents the mean ± SD with at least two independent experiments.

Mechanistic studies (Fig. 4C, Supplementary Fig. S1C) were performed to assess the inhibitory property of riboflavin on the Casp1 activity. The effects of riboflavin on the activation of other inflammasomes, such as AIM2, NLRC4, and non-canonical inflammasomes, were next examined^8,19^. As shown in Fig. 5A, LPS-primed BMDMs were introduced dsDNA, flagellin, or LPS into the cytosol to trigger AIM2, NLRC4, or non-canonical inflammasome assembly, and the IL-1β secretion was observed. Riboflavin disrupted the IL-1β released in response to the transfection of dsDNA, flagellin, and LPS. In addition, bacterial triggers, such as *Listeria monocytogenes* (*Listeria*), *Salmonella* Typhimurium (*Salmonella*), and *Escherichia coli* (*E. coli*), were used to activate inflammasomes. LPS-primed BMDMs were inoculated with the bacteria, and the levels of IL-1β secretion were measured (Fig. 5B). Consistent with the above data, riboflavin inhibited IL-1β secretion resulting from bacteria-mediated inflammasome activation. Finally, the inhibitory effects of riboflavin on the inflammasome activation in human macrophage-like cells, PMA-treated THP-1, were confirmed. As shown in Fig. 5C, riboflavin inhibited IL-1β secretion resulting from the activation of human inflammasomes. Therefore, riboflavin is a candidate for anti-inflammasome molecules.

## Discussion

This study examined the effects of vitamin B on the NLRP3 inflammasome activation. Among the B vitamins (B1, B2, B3, B5, and B6), riboflavin (B2) blocked IL-1β secretion, resulting from NLRP3 inflammasome activation. In addition, riboflavin inhibited the other readouts of inflammasome activation, such as the maturation of IL-1β, the secretion of Casp1 and IL-18, the formation of ASC pyroptosis, and the cleavage of Gsdmd. Riboflavin attenuated the secretion of IL-1β and IL-18 not only in macrophages but also in mice injected with MSU to trigger the NLRP3 inflammasome assembly. Mechanistically, riboflavin inhibited mtROS production and the release of mtDNA in response to an NLRP3 trigger. Although riboflavin did not alter the priming step of the NLRP3 inflammasome activation, it interrupted the activity of recombinant caspase-1. In addition, it was blocked by riboflavin, i.e., the activation of the AIM2, NLRC4, and caspase-11 inflammasomes, and bacterial inflammasome activation were also disrupted by riboflavin. In conclusion, riboflavin is a candidate to control inflammasome-mediated diseases.

Inflammasome is an intracellular multiprotein complex that consists of sensor proteins, inflammatory caspases, and adopter proteins^20^. The sensor proteins recognize the cytosolic endogenous or exogenous signals, and sequentially recruit caspase-1 with/without an adaptor protein^20^. Caspase-11 inflammasome also recruits caspase-1 by activating NLRP3 inflammasome^20^. Thus, the recruitment of caspase-1 of NLRP3, NLRC4, AIM2, and non-canonical inflammasomes is a common process that controls the maturation of IL-1β and IL-18 and the cleavage of Gsdmd. This study found that riboflavin inhibited the activity of recombinant caspase-1. Caspase-1 is produced as an inactive zymogen and consists of a catalytic domain and caspase recruitment domains (CARD). The recruited caspase-1 in the inflammasome assembly formed a dimer, p33 (CARD and p20) and p10, which binds to Asc and presents proteolytic activity^9^. p33 breaks down further to CARD and p20 and detaches from Asc^9^. The binding form of p20 and p10 is secreted, and the protease activity is terminated^9^. The activity of caspase-1 inhibited by CARD-only proteins (COPs), which has only CARD, not a catalytic domain, and by Ac-YVAD-cmk, Ac-YVAD-CHO, and belnacasan (VX-765)^9,21^. Based on Fig. 4C and Supplementary Fig. S2B, it was speculated that riboflavin inhibited the activity of caspase-1 resulting from other inflammasome activations, such as NLRC4, AIM2, and caspase-11. As shown in Fig. 5A,B, riboflavin attenuated the IL-1β secretion resulting from NLRP3, NLRC4, AIM2, and/or caspase-11 inflammasome activation. Although this study could not evaluate the selectivity or specificity of the inhibitory property of riboflavin on the activity of caspase-1, riboflavin was suggested to be an inhibitor of caspase-1.

Several studies have shown that riboflavin has an anti-inflammatory effect in animal models^5^. The intravenous administration of riboflavin presented the enhanced phagocytosis when tested with the isolated macrophages from the peritoneal cavity of mice^5^. In addition, the intramuscular injection of riboflavin into a cow increased the number of neutrophils and their phagocytosis^22^. The riboflavin treatment ameliorates the lethality of mice infected with several pathogens, such as *E. coli*, *Pseudomonas aeruginosa*, *Klebsiella pneumoniae*, *Staphylococcus aureus*, and *Actinobacillus pleuropneumoniae*^5,6,23^. In addition, the survival rates of mice injected with endotoxin (LPS) and exotoxin (*S. aureus* enterotoxin B) were also improved by the administration of riboflavin^5^. These protective effects on mortality were explained by the reduced pro-inflammatory cytokine production in the infected mice after the riboflavin injection. Indeed, the intravenous infusion of riboflavin into the sepsis mice alleviated the plasma level of pro-inflammatory mediators, such as TNFα, IL-1β, IL-6, INFγ, MCP1, and NO^5,6^. These studies, however, lacked mechanistic studies to account for the reduction of the pro-inflammatory cytokines when treated with riboflavin. Moreover, and the data were reported before the establishment of the inflammasome concept. TLR4 had been considered a gatekeeper of LPS-induced lethality (endotoxemia) and *E. coli*-induced septic shock, but the non-canonical inflammasome, mediated by caspase-11 in mice or caspase-4/5 in humans, is newly defined as the gatekeeper for the sepsis^19^. For example, mice infected with *E. coli* showed significantly low mortality in the deficiency of caspase-11^24^. Caspase-11 interacted directly with cytosolic LPS, leading to the Gsdmd cleavage, which formed the pyroptic pores, and triggered NLRP3 inflammasome assembly provoking IL-1β maturation via Casp1 activation^19^. Therefore, the protective effects of riboflavin on sepsis can be explained by the anti-inflammasome properties of riboflavin.

Zhang et al. reported that vitamin B6 inhibits NLRP3 inflammasome activation^25^. Vitamin B6 is a group of chemically similar compounds that are converted to an active form in biological systems. Pyridoxine (PN), pyrodoxal (PL), or pyridoxamine (PM) of vegetable or meat were absorbed in the intestine, and converted to pyrodoxal-5′-phosphate (PLP) by a kinase and oxidase^26^. PLP, the active form of vitamin B6, serves as a coenzyme in the enzyme reactions^26^. A previous study^25^ examined the effects of PN, PM, PL, and PLP on the activation of NLPR3 inflammasome in murine macrophages. As a result, PL and PLP significantly attenuated the IL-1β secretion resulting from NLRP3 inflammasome activation while PN and PM did not^25^. In Fig. 1, PN, which is the most common supplement of vitamin B6, was used to elucidate the anti-NLRP3 property. Similar to the previous study^25^, PN did not alter IL-1β secretion resulting from NLRP3 inflammasome activation.

## Materials and methods

### Cell preparation and culture

In the current study, two types of macrophages were utilized: bone marrow-derived macrophages (BMDMs) and human monocyte-like cell line (THP-1, Kore Cell Line Bank, Seoul, Republic of Korea)^27^. Unless indicated otherwise, the cell culture materials were purchased from Capricorn Scientific GmbH (Edsdorfergrund, Germany), and the plastics were provided from SPL Life Sciences (Gyeonggi-do, Republic of Korea). To prepare the BMDMs, myeloid cells were collected from mice (C57BL/6, 6 to 12 weeks old, Narabio Co., Seoul, Republic of Korea), and differentiated to macrophages under L929 cell-conditioned media (LCCM) to supply macrophage colony-stimulating factors^28^. The progenitors were cultured for seven days in RPMI 1640 media containing 10% fetal bovine serum (FBS), 30% LCCM, and antibiotics. THP-1 cells were cultured in RPMI 1640 media containing 10% FBS and antibiotics and differentiated to macrophage-like cells by a treatment of phorbol 12-myristate 13-acetate (200 nM, PMA; InvivoGen, San Diego, CA, USA) for 24 h. All cells were incubated at 37 °C in an atmosphere containing 5% CO_2_.

### Inflammasome activation

As shown in Fig. 1A, BMDMs or PMA-treated THP-1 cells (1 × 10^6^ cells per well, 12-well plate) were seeded and treated with lipopolysaccharides (LPS, 1 μg/ml; Sigma-Aldrich Co., St. Louis, MO, USA) for 3 h. These LPS-priming cells were treated further with inflammasome triggers in the presence of vitamins. For the screening test in Fig. 1, vitamin B1 (thiamine hydrochloride), B2 (riboflavin), B3 (niacinamide), B5 (calcium pantothenate), B6 (pyridoxine hydrochloride), and C (ascorbic acid) were purchased from ES food Co. (Gyeonggi-do, Republic of Korea). The riboflavin was also provided by Sigma-Aldrich Co. and Daejung Chemicals & Metals Co. LTD. (Daejeon, Republic of Korea). To activate the inflammasomes, the LPS-primed macrophages were subjected to RPMI 1640 media (350 μl) containing a inflammasome trigger as follows: nigericin (NG, 40 μM, Tocris Bioscience, Bristol, UK) for 1 h, adenosine triphosphate (ATP, 5 mM, InvivoGen) for 1 h, monosodium urate crystals (MSU, 200 μg/ml, U2875, Sigma–Aldrich Co.) prepared using previously reported methods^29^ for 3 h, rotenone (160 μM, Santa Cruz Biotechnology, Dallas, TX, USA) with/without diphenyleneiodonium (DPI, 200 μM, Tocris Bioscience) for 6 h, flagellin^30^ (500 ng/ml, InvivoGen) with Lipofectamine 2000 (10 μl/ml, Invitrogen, Carlsbad, CA, USA) for 1 h, double-stranded DNA (1 μg/ml, dsDNA) with jetPRIME (2 μl/ml, Polyplus-transfection Inc., Illkirch, France) for 1 h, LPS (15 μg/ml, Sigma-Aldrich Co) with jetPRIME (2 μl/ml, Polyplus-transfection Inc.) for 6 h, *Salmonella enterica* serovar Typhimurium^31^ (*Salmonella*, multiplicity of infection [MOI] 3.5) for 1 h, *Listeria monocytogenes*^31^ (*Listeria*, MOI 35) for 6 h, and *Escherichia coli*^32^ (*E. coli*, MOI 10, DH5α, Invitrogen, CA, USA) for 6 h. The above bacteria were grown at 37 °C on Luria–Bertani media (Condalab, Torrejón de Ardoz, Madrid, Spain) for *Salmonella* and *E. coli*, or Brain Heart Infusion media (Condalab) for *Listeria*.

### Immunoblot assay

The samples for immunoblotting were prepared as follows. The cellular supernatants (Sup, 200 μl) were harvested from the 12-well plates after inflammasome activation, and the cells were lysed with a mild lysis buffer (150 mM NaCl, 1% Triton X-100, 50 mM Tri-base, pH 8.0) containing a Halt proteinase inhibitor cocktail (ThermoFisher Scientific, Waltham, MA, USA). The cellular lysate (Lys, 100 μl) was collected after centrifugation (15,000 rcf, 5 min), and the remaining debris were cross-linked with suberic acid bis (2 mM, Sigma-Aldrich Co.) for 1 h. The pellet after centrifugation (15,000 rcf, 5 min) were suspended in 50 μl of loading dye buffer (116 mM Tris, 3.4% SDS, 12% glycerol, 200 mM DTT, 0.003% bromophenol blue).

The Sup, Lys, and pellet were electrophoresed by SDS-PAGE (10 or 16%) and transferred to a membrane (PVDF, Pall Co., Port Washington, NY, USA) using Mini-PROTEAN Tetra Handcast Systems and Criterion Blotter (BIO-RAD, Hercules, CA, USA) according to the manufacturer’s protocol. The membrane was primary probed overnight with the following anti-sera at 4 °C: anti-mouse IL-1β antibody (AF-401-NA, R&D Systems, Minneapolis, MN, USA), anti-Caspase-1(p20) antibody (AG-20B-0042-C100, AdipoGen Co., San Diego, CA, USA), anti-Asc antibody (sc-22514, Santa Cruz Biotechnology), anti-GSDMD antibody (ab209845, Abcam plc., Cambridge, MA, USA), or anti-Actin antibody (sc-1615, Santa Cruz Biotechnology). After primary probing, the membranes were incubated further with the second antisera conjugated with HPR (Santa Cruz Biotechnology) for 3 h at room temperature. Finally, the membranes were visualized using a chemiluminescence solution (WESTSAVE STAR, AbFrontier, Seoul, Republic of Korea) and a chemiluminometer (EZ-Capture II, ATTO Technology, Tokyo, Japan). Full-length bolts for figures were shown in Supplementary Information.

### Animal experiments

C57BL/6 mice (female, 7 weeks old, Narabio. Co) were maintained under a 12 h light/dark cycle at 18 to 24 °C and given standard chow and tap water ad libitum. After one week of acclimation, the mice were administered riboflavin (Sigma-Aldrich Co. or Daejung Chemicals & Metals Co. LTD.) by oral gavage or intra-peritoneal (IP) injection before or after MSU crystal (5 mg/mouse, IP) injection, as illustrated in Fig. 3B. The mice were sacrificed by CO_2_ inhalation 6 h after the MSU injection, and the peritoneal lavages were collected by washing with 5 ml PBS. The mouse experiments in the current study were conducted under the National Institutes of Health Guide for the Care and Using of Laboratory Animals and approved by the Institutional Animal Care and Use Committee of Kangwon National University (approval no. KW-200210-2).

### Assays for cytokines, Casp1 activity, cytotoxicity, and cytosolic mitochondrial DNA

The secretion of IL-1β or IL-18 in the cellular supernatant and the peritoneal lavage was analyzed by mouse IL-1beta/IL-1F2 ELISA kit (DY401, R&D Systems), or home-made ELISA plates using the mouse IL-18/IL-1F4 antibody (D047-3, R&D Systems) and mouse IL-18/IL-1F4 biotinylated antibody (D048-6, R&D Systems). In addition, the activity of casp1 was examined using a Caspase-Glo 1 inflammasome assay (Promega Co., Madison, WI, USA) and human recombinant caspase-1 (hrCasp1, one unit per reaction, Enzo Biochem, Inc., Farmingdale, NY, USA). For the cytotoxicity assay, BMDMs (5 × 10^4^ cells per well in a 96-well plate) were treated with a vitamin, as indicated overnight, and the cell viability was measured by EZ-Cytox (DoGen Bio, Seoul, Republic of Korea) according to the manufacturer’s protocol^33^. These assays were analyzed by a microplate reader (Synergy H1 microplate reader, BioTek, Winooski, VT, USA).

To measure the release of mitochondrial DNA, the LPS-primed BMDMs (2 × 10^6^ cells per well in 6-well plate) were treated with ATP (5 mM) for 1 h, and then lysed with IGEPAL CA-630 (1%, Sigma–Aldrich Co.) for 15 min on ice. The cytosolic fraction was prepared by centrifugation at 15,000 rcf for 30 min at 4 °C, and the cytosolic DNA was harvested using a G-spin total DNA extraction kit (Intron Biotechnology, Gyeonggi-do, Republic of Korea)^34^. The copy number of cytochrome c oxidase 1 and 18S rDNA were quantified by real-time PCR using TOPreal qPCR 2 × PreMIX (Enzynomics Co., Daejeon, Korea) and the following specific primers^35^: 18 s rDNA, 5′-TAG AGG GAC AAG TGG CGT TC′ and 5′-CGC TGA GCC AGT CAG TGT-3′; cytochrome c oxidase 1, 5′-GCC CCA GAT ATA GCA TTC CC-3′ and 5′-GTT CAT CCT GTT CCT GCT CC-3′.

### Statistics

For statistical analyses, two or more groups were analyzed using a t-test (Mann–Whitney test) or one-way ANOVA (Tukey’s multiple comparisons test) using GraphPad Prism 6 (GraphPad Software, San Diego CA). The P values are indicated in the figures.

## Supplementary information

Supplementary Information.
