# Supplementary material for: Riboflavin, vitamin B2, attenuates NLRP3, NLRC4, AIM2, and non-canonical inflammasomes by the inhibition of caspase-1 activity
Source: Sci Rep. 2020 Nov 5;10:19091. doi: 10.1038/s41598-020-76251-7 (PMC7645791; doi:10.1038/s41598-020-76251-7)

Supplementary Information

Riboflavin, vitamin B2, attenuates NLRP3, NLRC4, AIM2, and non-canonical inflammasomes by the inhibition of caspase-1 activity

Huijeong Ahn, and Geun-Shik Lee*

College of Veterinary Medicine and Institute of Veterinary Science, Kangwon National University, Chuncheon, Gangwon, 24341, Republic of Korea.

*Correspondence: Geun-Shik Lee, D. V. M., Ph. D.

Laboratory of Inflammatory Diseases, Department of Physiology, College of Veterinary Medicine, Kangwon National University, Chuncheon, Gangwon, 24341, Republic of Korea.

e-mail: leegeun@kangwon.ac.kr, Tel: +82-33-250-8683, Fax: +82-33-244-2367

Supplementary figure 1.

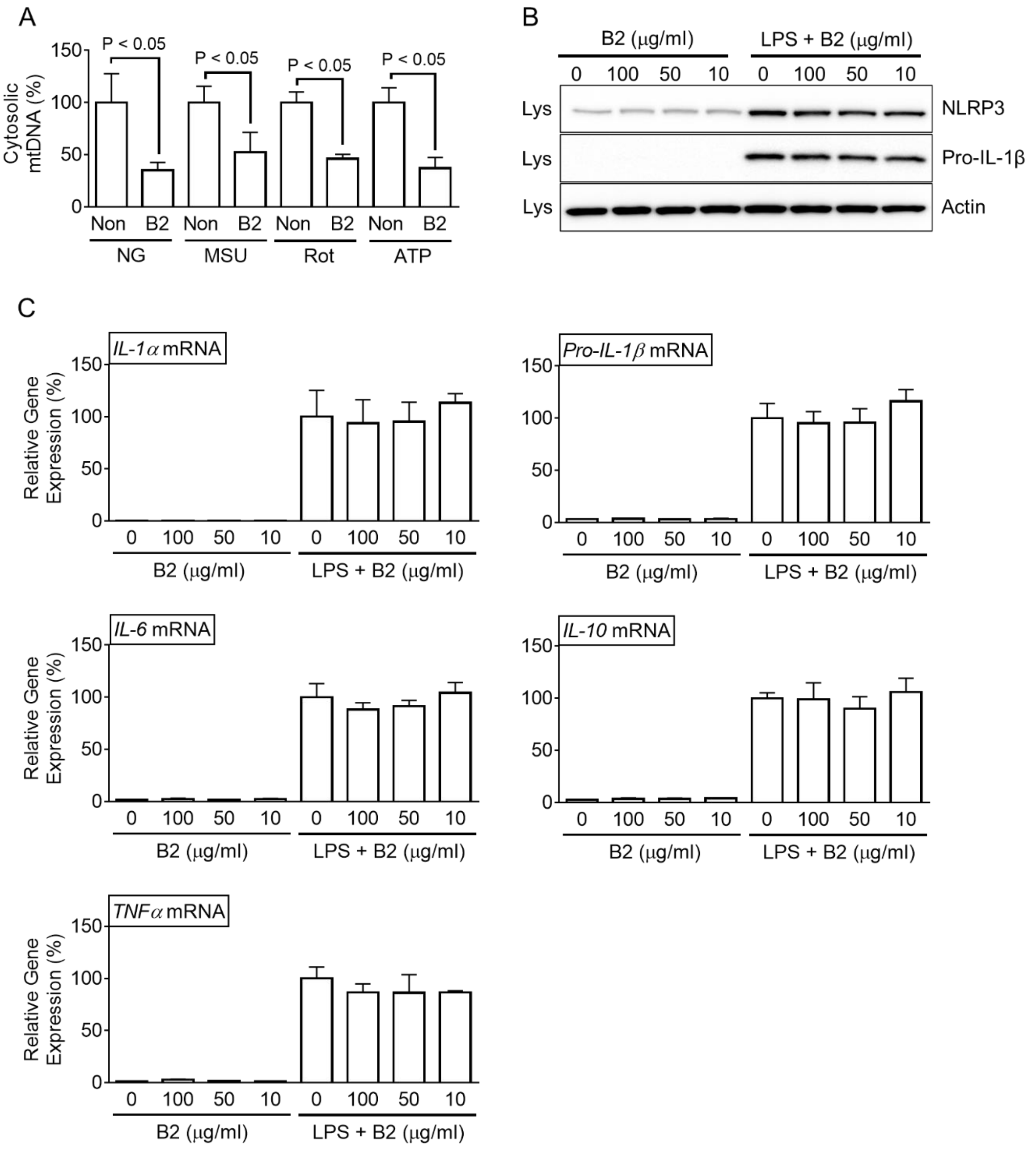

Supplemental figure 1. Effects of riboflavin on the cytosolic release of mtDNA, the priming of inflammasome activation, and the transcription of cytokines

A, LPS-primed BMDMs (2×10^6 cells per well in 6-well-plate) were treated with NG (40 μM), MSU (400 μg/ml), rotenone (Rot, 160 μM), or ATP (2mM) with/without riboflavlin (B2, 100

μg/ml) for 1h. The cytosolic release of mitochondrial DNA (mtDNA, cytochrome c oxygenase 1 / 18S rDNA) was measured by quantitative real-time PCR. **B**, BMDMs (2×10^6 cells per well in 6-well-plate) were treated with LPS (10 ng/ml) for 3h. The protein expression of NLRP3 (~118 kDa) and pro-form of IL-1β (pro-IL-1β, ~37 kDa) were detected by immunoblotting. **C**, The total RNA was isolated from the same experiment of panel A using NucleoZOL (MACHEREY-NAGEL GmbH & Co. KG, Postfach, Düren, Germany) and synthesized into first-strand complementary DNA (cDNA) using a random primer and reverse transcriptase (M-MLV RT, Enzynomics Co., Daejeon, Korea) according to the manufacturer's protocol. The copy number of cytokines were measured using qPCR premix (TOPreal™ qPCR 2× PreMIX, Enzynomics Co.), a real-time PCR machine (Eco Real-Time PCR system Illumina, San Diego, CA, USA), and specific primers as follows: *IL-1α* (Gene Bank ID: NM_010554) 5′-CCG ACC TCA TTT TCT TCT GG-3′ and 5′-GTG CAC CCG ACT TTG TTC TT-3′; *Pro-IL-1β* (NM_008361) 5′-CCC AAG CAA TAC CCA AAG AA-3′ and 5′-GCT TGT GCT CTG CTT GTG AG-3′; *IL-6* (NM_031168) 5′-CCG GAG AGG AGA CTT CAC AG-3′ and 5′-TCC ACG ATT TCC CAG AGA AC-3′; *IL-10* (NM_010548) 5'-TCA TTT CCG ATA AGG CTT GG-3' and 5'-TGC TAT GCT GCC TGC TCT TA-3'; *TNFα* (NM_013693) 5′-ACG GCA TGG ATC TCA AAG AC-3′ and 5′-GTG GGT GAG GAG CAC GTA GT-3′; GAPDH (*Gapdh*; NM_001289726) 5'-AAC TTT GGC ATT GTG GAA GG-3' and 5'-ACA CAT TGG GGG TAG GAA CA-3'. **C**, The effects of B2 on the activity of mouse recombinant caspase-1 (mrCasp1, Enzo Biochem Inc.) was analyzed using a Caspase-Glo®1 inflammasome assay (Promega Co.). The bar graph presents the mean ± SD with at least two independent experiments.

Supplementary figure 2.

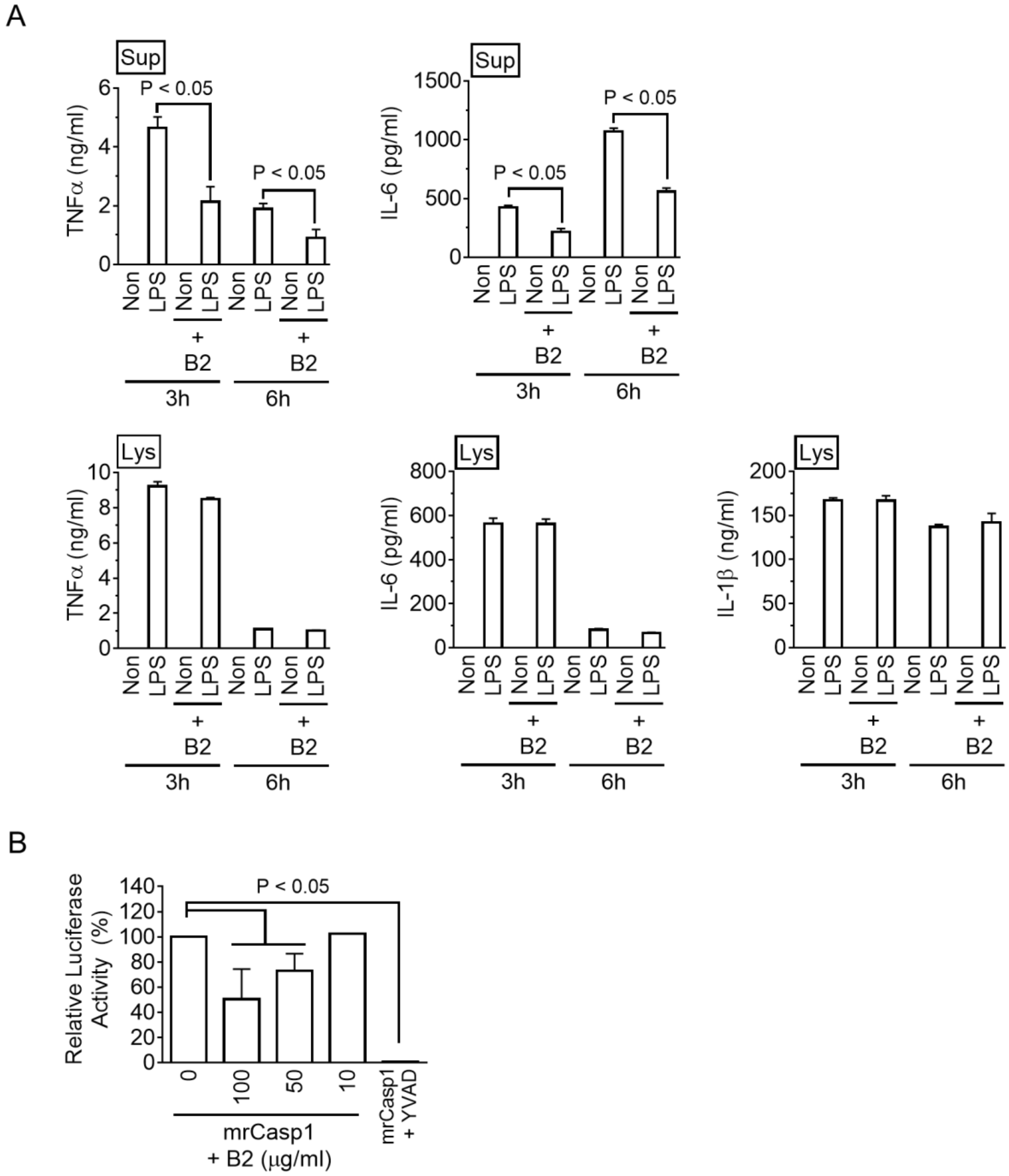

Supplemental figure 2. Effects of riboflavin on the secretion and production of TNFα and IL-6, and the activity of mouse recombinant caspase-1

A, BMDMs (1 × 10^6 cells per well in 12-well-plate) were treated with LPS (10 ng/ml)

with/without riboflavin (B2, 100 μg/ml) for 3h or 6h. The levels of TNFα and IL-6 in the cellular supernatants (Sup) or lysates (Lys) were detected by ELISA. **B**, The effects of B2 on the activity of mouse recombinant caspase-1 (mrCasp1, Enzo Biochem Inc.) was analyzed using a Caspase-Glo®1 inflammasome assay (Promega Co.). The bar graph presents the mean ± SD with at least two independent experiments.

Full-length blots of Figure 2A

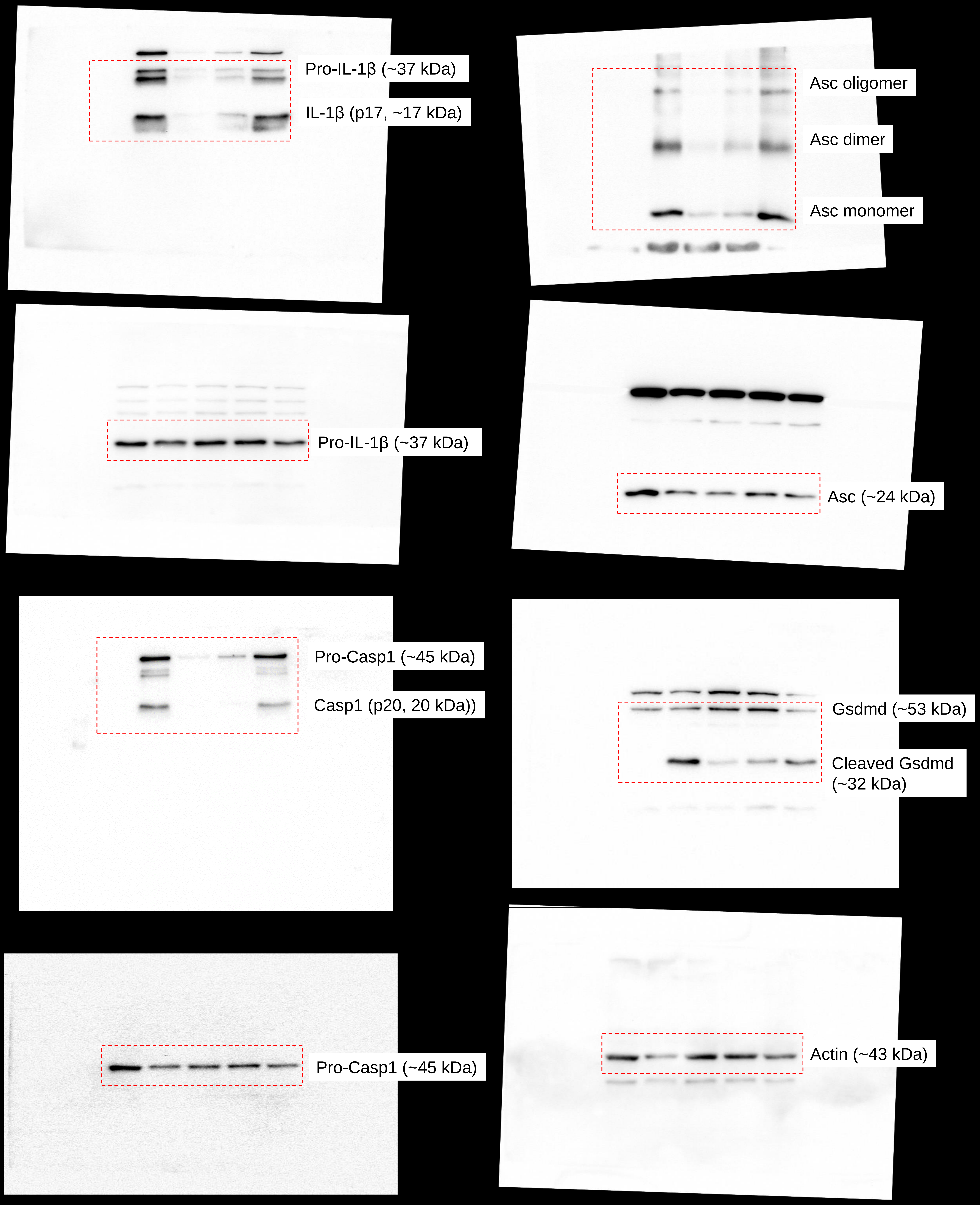

Full-length blots of Figure 2C

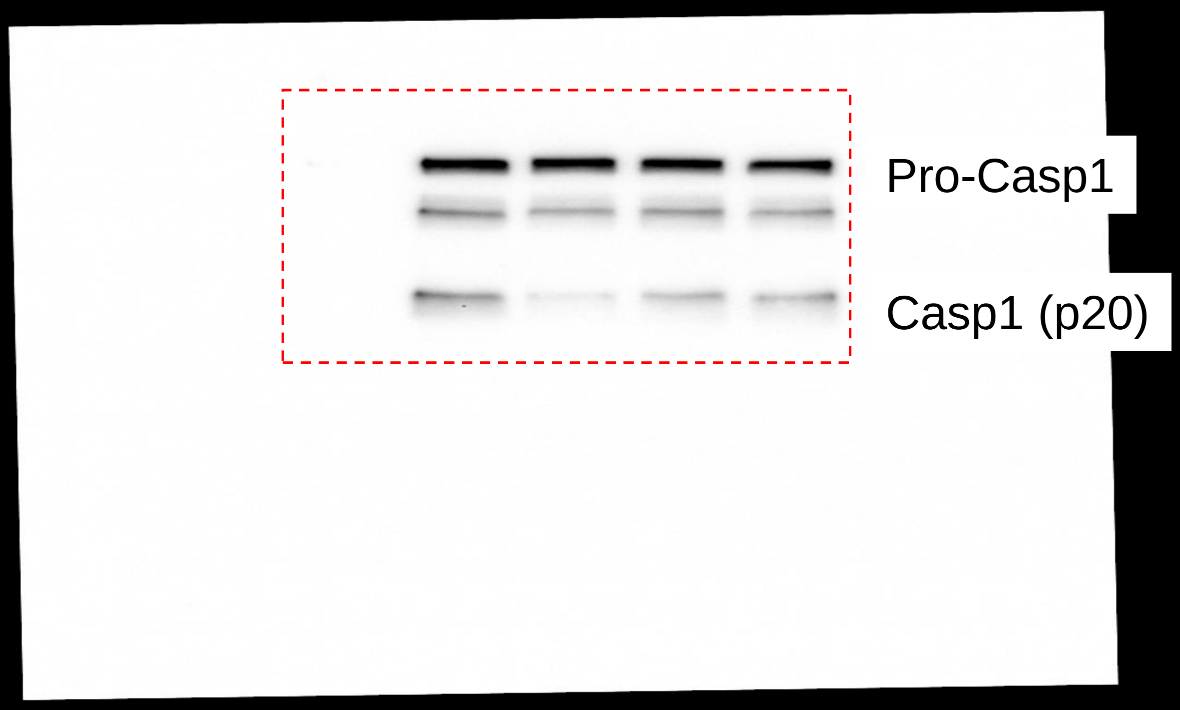

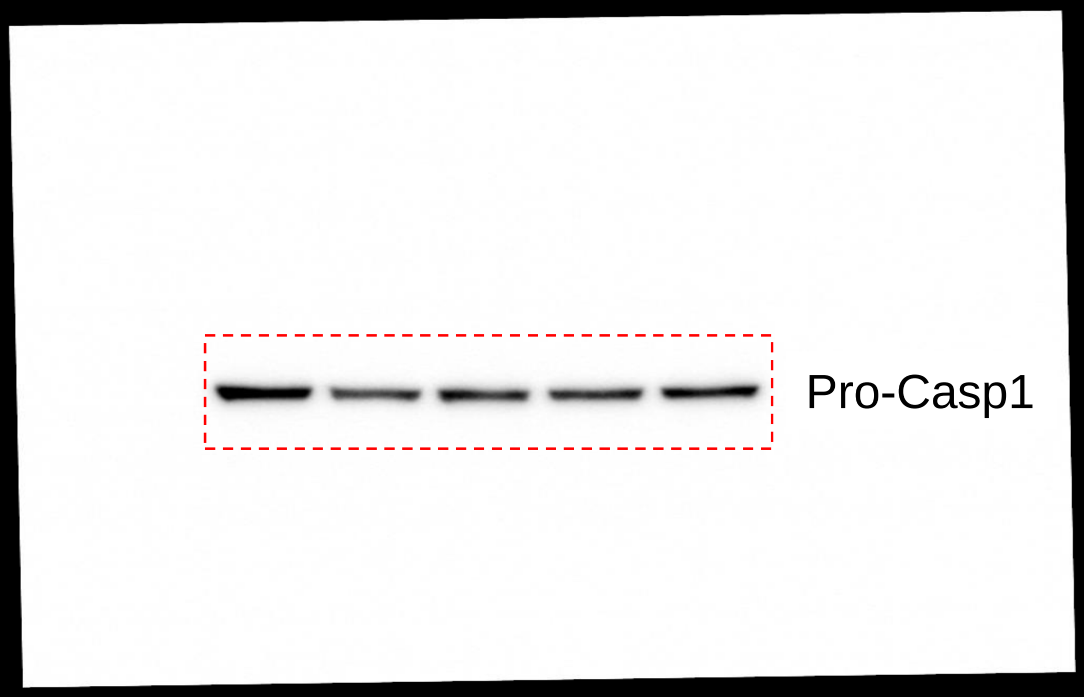

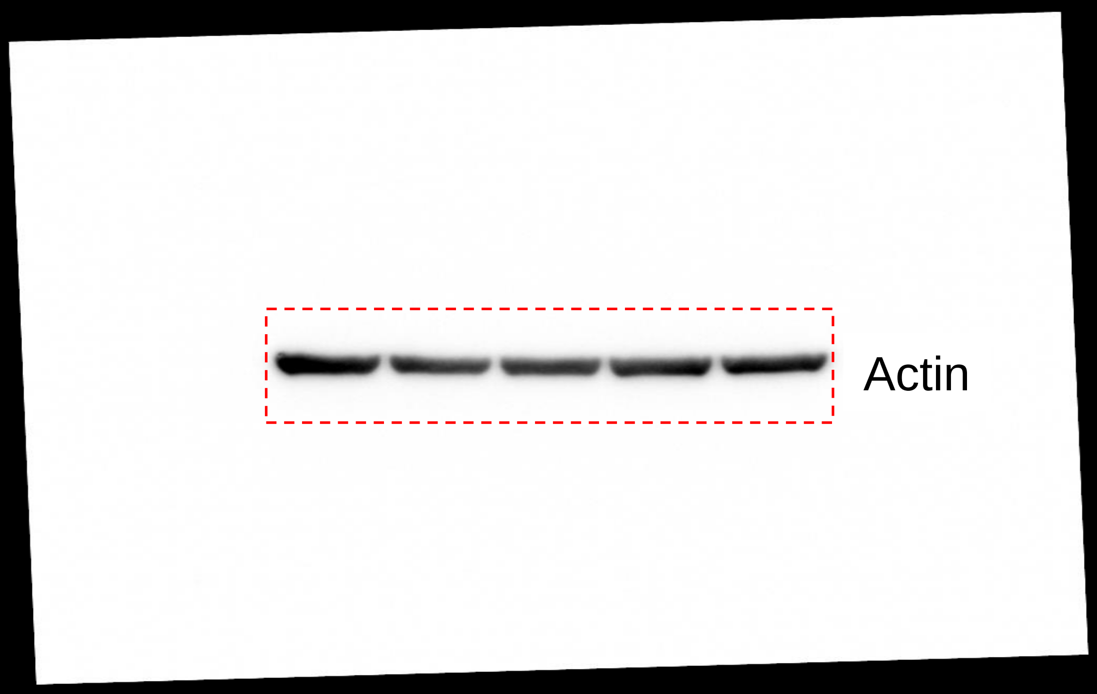

Full-length blots of Figure 3A

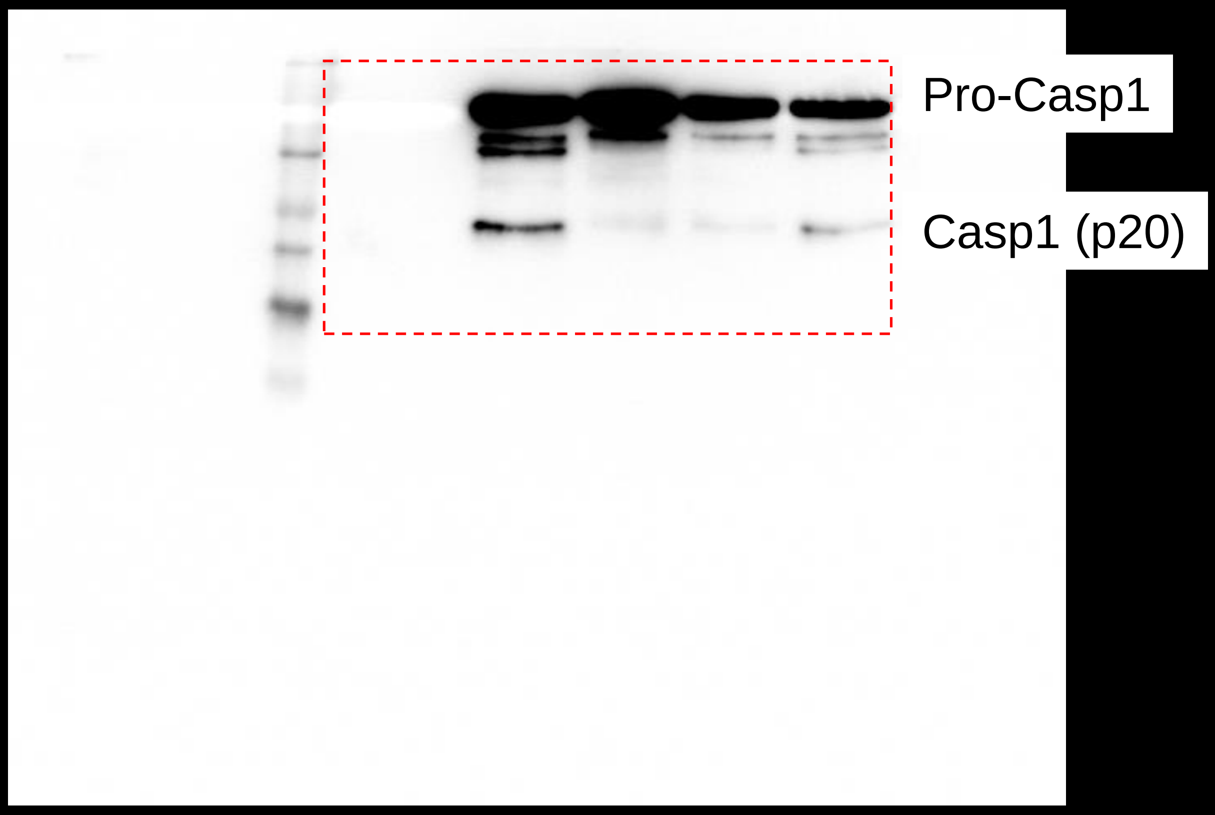

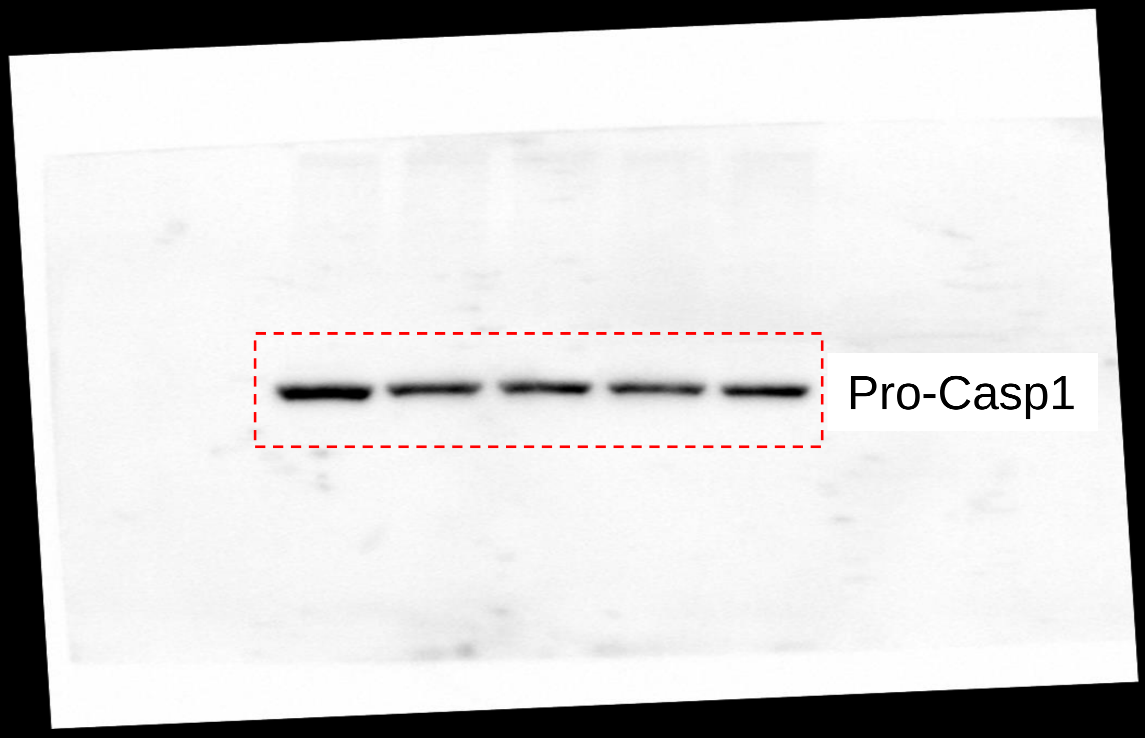

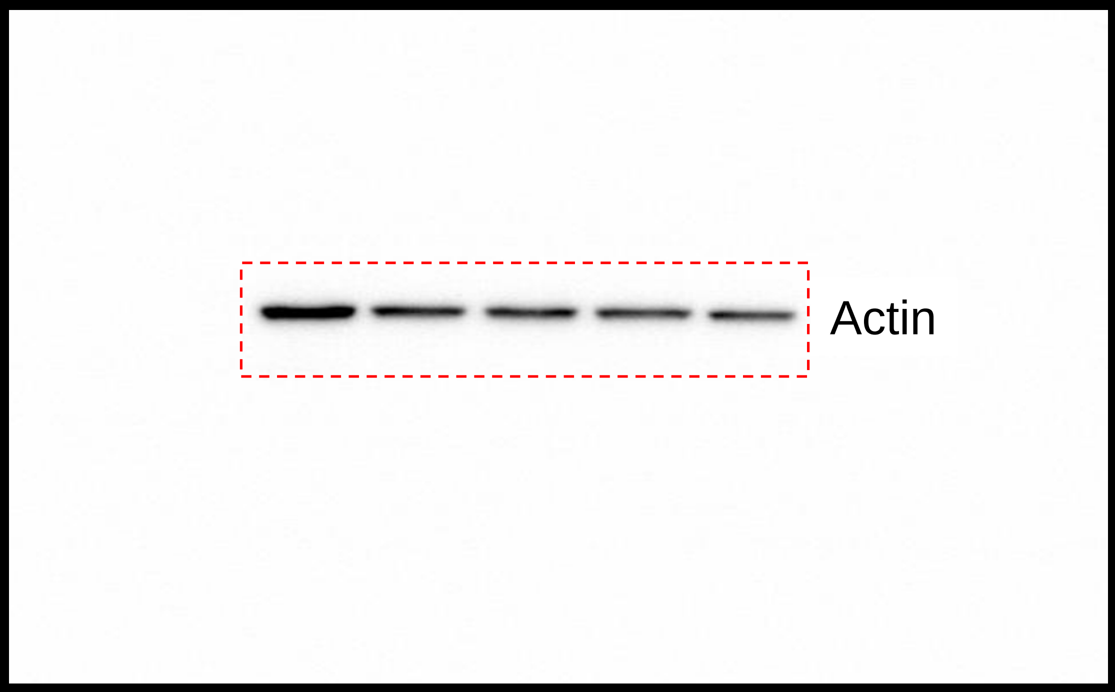

Full-length blots of Figure 4A

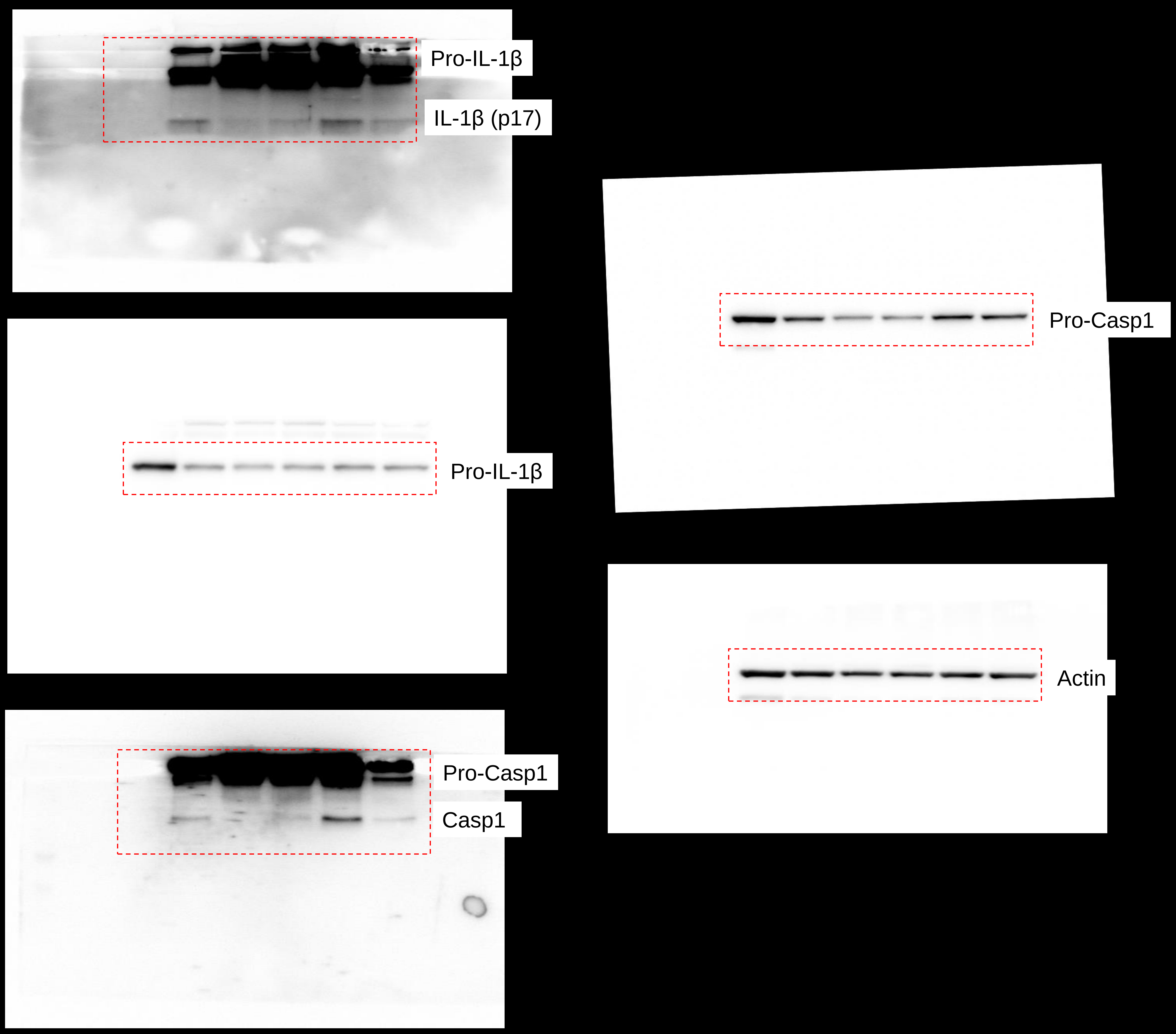

Supplement: Supplementary file 1 — Supplementary Information. [file 41598_2020_76251_MOESM1_ESM.pdf]
